# A brief history of interventional radiology in Singapore and its current status

**DOI:** 10.2349/biij.7.2.e13

**Published:** 2011-04-01

**Authors:** TKB Teo, BS Tan, KH Tay

**Affiliations:** Department of Diagnostic Radiology, Singapore General Hospital, Singapore

**Keywords:** Interventional radiology, Singapore radiology

## Abstract

X-ray services were first established in Singapore in 1898. With the opening of the General Hospital in 1926, there was subsequent increase in workload. However, a radiology department was not formed until the 1950s. Angiography was introduced in the same decade initially for diagnosis. By the 1960s and 1970s, both vascular and non-vascular interventions were performed. Subsequently, interventional radiology experienced exponential growth, with newer technology and better facilities established over the past 3 decades. With more trained interventional radiologists, the service is currently available in all public hospitals and in most private hospitals in Singapore today. It is envisaged that structured training and formal credentialing will be established, eventually leading to recognition of interventional radiology as a specialty in its own right.

## INTRODUCTION

The tremendous global expansion of interventional radiology over the past 3 decades has been mirrored in the development of this specialty in Singapore. For many conditions, interventional radiology techniques are now the preferred treatment of choice. However, as it is a young specialty, there are challenges of recognition by the public and within the medical profession. To understand the current status of interventional radiology in Singapore, it is critical that we review its history.

## HISTORY OF INTERVENTIONAL RADIOLOGICAL PRACTICE AND EARLY PIONEERS

The use of X-ray reached Singapore shores in January 1898, only three short years after Wilhelm C. Roentgen’s discovery. It was a small X-ray unit installed at the Municipal Office, battery operated and took seven minutes to obtain an exposure. In 1913, an X-ray apparatus was installed in the General Hospital but it was not functional due to dampness. A Coolidge tube was later installed in 1920 which improved imaging considerably [1].

As work increased exponentially over the years, it was felt that a full-time X-ray specialist was needed. In 1925, Dr. JS Webster, who was a Professor of Medicine at that time was designated Radiologist, General Hospital [2]. From the opening of the new General Hospital in 1926 to the pre-war period, the annual number of X-ray examinations and radiotherapy procedures grew at an accelerated pace. Medical students were even taught a course in radiology and roentgenotherapy in 1934, and in 1937, a radiological anatomy course was introduced [1].

After the war, there was increased staffing and volume of work, and this culminated in the development of the radiology department in the 1950s. At this time, both diagnostic and therapeutic radiology were under the same umbrella but this soon split into two different departments in 1968 [1].

**Figure 1:**
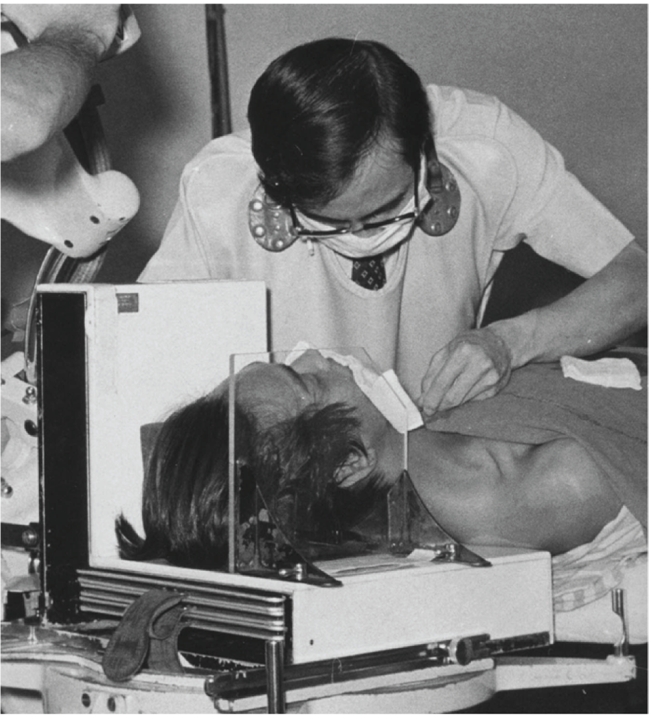
Dr. Eric Wong performing a cerebral angiogram using direct puncture of the left carotid artery, on a skull unit (Elema Schonander) with cassette changer (Bar & Stroud).

Angiography started in Singapore in the mid-1950s. This was performed by direct needle puncture and injection (Figure 1). Not surprisingly, this was started by physicians and surgeons with special interests in neurology, cardiology and vascular surgery, as there was a shortage of radiologists at this time [1]. Dr. TJ Danaraj, Dr. Gwee Ah Leng, Dr. Khoo Oon Teik and Dr. Yong Nen Khiong were some of the notable figures performing the procedure. As would be expected, these procedures were dangerous with inconsistent results and poor quality images. In 1959, Dr. Chow Khuen Wai introduced the safer and more versatile Seldinger technique of vascular catheterization upon his return from the United Kingdom after further training. This also coincided with the installation of an AOT automatic cassette serial changer. By then, all angiographic work was undertaken by radiologists. In 1963, a 1000 mA unit for cardiovascular work was installed in Singapore General Hospital, and this was subsequently coupled to an image intensifier.

By the 1960s, radiology began its transition from a paraclinical specialty to a clinical one [3]. In 1964, Dr. Chow returned from a six-month WHO fellowship in cardiovascular radiology and development gained further momentum under his direction. In the 1960s and 1970s, interventional radiology expanded into both vascular and non-vascular procedures. Selective angiography, which was first introduced in the early 1960s, continued to expand. Superselective catheterization was practiced from the 1970s as a result of improvement in materials, catheter design and operator skills [1].

**Figure 2:**
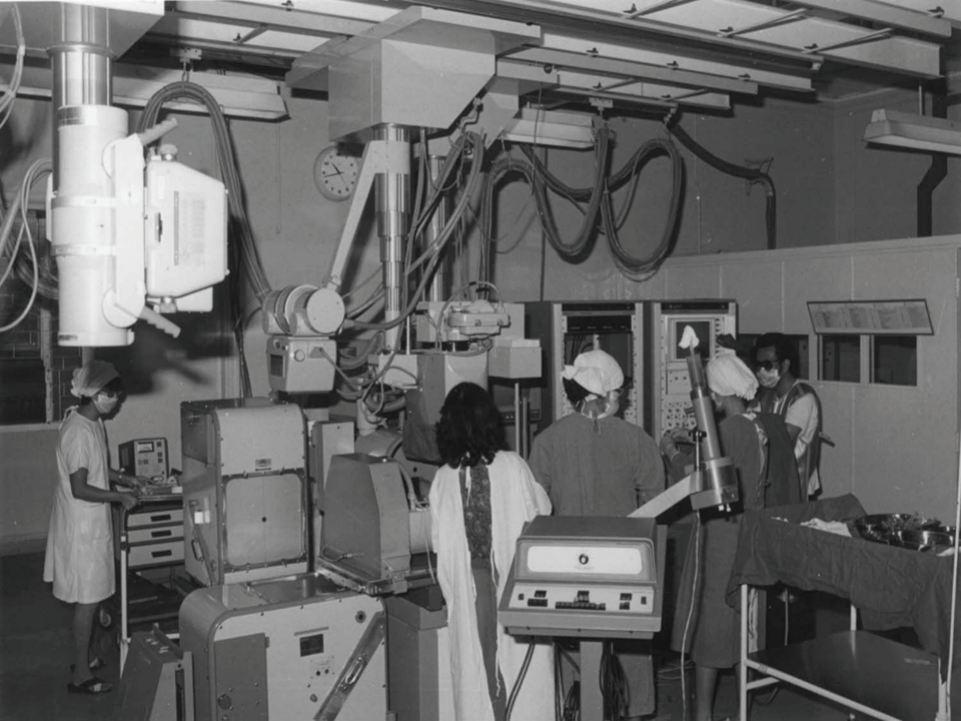
Room F in the Singapore General Hospital was a Cardio-angiography room in the 1970s.

In the early 1970s, the Ministry of Health committed to upgrading selected medical specialties, particularly neurosurgery and cardiovascular surgery. In 1975, to support the development of neurosurgery, a fully equipped neuroradiology suite was built in Tan Tock Seng Hospital. The equipment installed included the Mimer unit for air encephalography and ventriculography, and units for cerebral angiography and stereotaxy. To support cardiovascular surgery, in 1973, a bi-plane cine angiography unit was installed in the cardiovascular laboratory of Singapore General Hospital with the help of Dr. Audrey Pitt of the Royal Prince Alfred Hospital, Melbourne (Figure 2) [4]. For some time, catheters had to be fashioned by the radiologists for different angiographic procedures [3]. The first case of embolization for massive gastrointestinal bleeding was performed in 1976 by Dr. Boey Hong Khim [4]. Fine-needle aspiration biopsy with Chiba needles started in 1979 with needles brought back to Singapore from Japan [3].

Dr. Lenny Tan pursued a fellowship in interventional radiology in the United States in the late 1970s and on his return introduced many more new techniques, bringing interventional radiology in Singapore to a higher plane.

**Figure 3:**
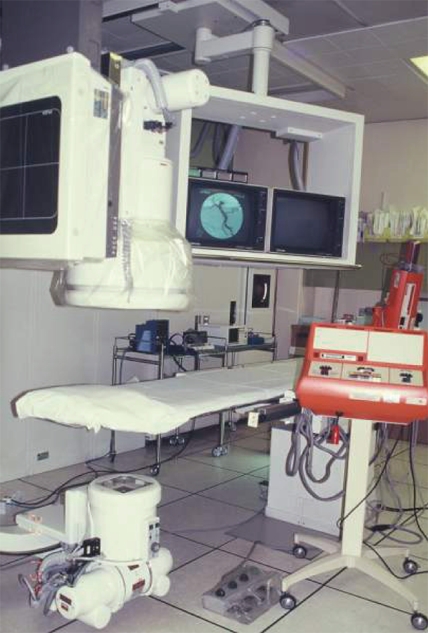
Manual subtraction angiography unit using automated cassette changer (Toshiba) in use from 1988 to 1996 in the Singapore General Hospital.

In the meantime, with the redevelopment and building of the many public hospitals during the 1980s and 1990s, angiographic facilities were planned for (Figure 3). The National University Hospital was opened in 1985, and interventional radiology services were made available. The Toa Payoh Hospital had a new fluoroscopy unit with closed-circuit television installed in 1981 and more fluoroscopic interventional procedures could be performed. However, it was not until its move in 1997 to the eastern part of the island, and after renamed as Changi General Hospital, that an angiographic unit was installed. Similarly, when the Kandang Kerbau Women’s and Children’s Hospital moved into new premises in 1997, angiographic facilities were installed.

Interventional radiology services were also established in the private hospitals during this time, notably at Mt Elizabeth Hospital and Gleneagles Hospital. Mt Elizabeth Hospital opened its doors in 1979, and from the early 1980s offered angiographic services. A cardiovascular laboratory was set up at the Gleneagles Hospital in 1988.

During this period of rapid growth, the contributions of numerous radiologists to the further development of interventional radiology including Drs Boey Hong Khim, Yaswant Patel, Eric Wong, Tan Kim Ping, Khoo Teng Kew, Austin Htoo, Low Cheng Hoon, Seetoh Cheong Wah, Edwin Siew, Robert Kwok, John Hoe, Thomas Chee, Cheong Wing Yee and Yeong Kuan Yuen should be acknowledged. These radiologists introduced and established many new interventional procedures.

The next step in development in interventional radiology was in sub-specialization. Dr. Francis Hui embarked on a fellowship in interventional neuroradiology under the guidance of the late Professor Pierre Lasjaunias in Paris during the early 1990s, and on his return introduced complex procedures like intracranial aneurysm and arteriovenous malformation embolization. This ushered in another era of expansion whereby many young and enthusiastic radiologists were sent to overseas centres of excellence to train in various areas of interventional radiology. They, in turn, contributed to the further development of the specialty after their return.

## CURRENT STATUS

**Figure 4:**
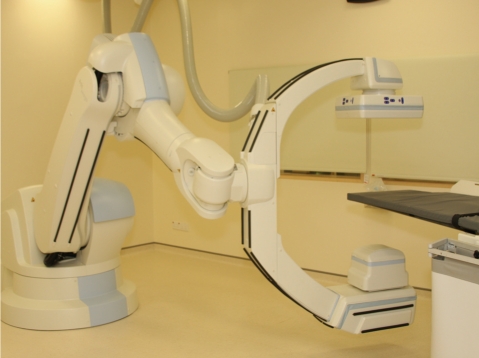
Current state-of-the-art flat panel digital angiography unit with cone-beam CT capability (Siemens) in use from 2008 to present in the Singapore General Hospital.

Today, every public hospital in Singapore has an interventional radiology service, with Tan Tock Seng Hospital, National University Hospital and Singapore General Hospital performing the most number of cases annually. In these hospitals, the interventional radiology service is provided by dedicated interventional radiologists who are also required to perform diagnostic radiology service work on several sessions per week. Because the case load in the other public hospitals is lower, interventional radiologists are expected to undertake more diagnostic work compared to interventional radiology procedures. The equipment available in all the public hospitals is state-of-the-art and the radiologists are supported by dedicated paramedical staff (Figure 4). Several of the private hospitals like Mt Elizabeth Hospital, Gleneagles Hospital and Mt Alvernia Hospital have also established interventional radiology services.

With these services available, the number of cases over the past decade has grown exponentially. In some of the services, interventional radiologists have dedicated clinics where patients are seen prior to the procedures and followed up. In some services, regular ward rounds are also performed to review patients and many interventional radiologists have admitting rights in their respective hospital. However, these practices are not standardised across all the services.

A Cardiovascular and Interventional Radiology subsection, under the umbrella of the Singapore Radiological Society has been formed and members meet regularly for case discussions.

## TRAINING AND CREDENTIALING

Despite increasing recognition that interventional radiology is a very specialised branch of medicine, it is not yet recognised as a specialty in Singapore. Credentialing for procedures is strict, with a requirement for a minimal number of cases performed under supervision before one can be allowed to perform them independently. However, credentialing is currently not national but hospital-based depending on each individual institution’s unique case-mix and workload.

All aspiring interventional radiologists have to complete three years of basic specialty training in diagnostic radiology. After passing the local Master of Medicine (Diagnostic Radiology) degree or admission as a Fellow of the Royal College of Radiologists (United Kingdom) or its equivalent, trainees undergo a further two years of advanced specialty training of three-monthly postings in various subspecialties. Although not cast in stone, it is an unspoken rule that the advanced specialty trainee will spend two of these three-monthly postings in their subspecialty of choice. After satisfactorily completing these two years of advanced training, the trainee is considered to have exited and met the statutory requirements for specialist registration as a diagnostic radiologist in Singapore. Hence, the minimal training to be a radiologist in Singapore requires five years of supervised training in an approved institution.

Therefore, the expected time that an aspiring interventional radiologist will spend in interventional radiology in his five years of formal radiology training is only six months. This is far from ideal as a newly minted consultant radiologist is expected to hit the ground running. In addition, the current model incorporates little structured training in clinical care, a component that will increasingly become more critical for practitioners of this specialty.

However, the above situation is not unique to Singapore. At the recent Society of Interventional Radiology (SIR) Annual Scientific Meeting 2009, the 2009 Dotter lecturer, Dr. Matthew A. Mauro spoke on ‘The Birth of a Specialty – Interventional Radiology’. In the United States, trainees undergo a four-year residency programme in diagnostic radiology before undergoing a one-year fellowship in interventional radiology. Similarly, the feeling amongst interventional radiologists is that one year is insufficient for training to be an attending/consultant. He detailed various schemes implemented. For example, he spoke of two-year diagnostic radiology and three-year interventional radiology programmes resulting in certification as an interventional radiologist, as well as three-year diagnostic radiology and three-year interventional radiology programmes resulting in duo certification in both. Surprisingly, the biggest opponents to these alternative training routes were from the diagnostic radiologists! The SIR has recently submitted a proposal to the American Board of Medical Specialties for recognition of interventional radiology as a distinct specialty in its own right.

## CONCLUSION

Interventional radiology in Singapore has seen tremendous growth over the past 50 years. It is perhaps prescient that Dr. FY Khoo, one of the early local Singapore radiologists, predicted that interventional radiology will enjoy ‘a period of stimulating challenges and, hopefully, of gratifying involvement’ in an article published in 1982 [1]. Indeed today, there is a critical mass of trained interventional radiologists practicing across the country, with access to state of the art equipment. Patients, when informed of the possibility of utilising minimally invasive techniques to treat their disease, invariably opt for them. With this growth in popularity, some of our clinical colleagues also now see learning interventional radiology techniques as desirable.

In the next few years, there is an urgent need for interventional radiologists to rise to meet the challenges of clinical demand, training and ‘tuft’. If these issues are not adequately addressed, even as more and more minimally invasive imaging guided techniques develop, interventional radiologists in Singapore may find that they are not the ones performing them.
